# Parent-Infant Interaction Tasks Adapted for Remote Testing: Strengths, Challenges, and Recommendations

**DOI:** 10.3389/fpsyg.2021.733275

**Published:** 2021-10-13

**Authors:** Shira C. Segal, Margaret C. Moulson

**Affiliations:** Department of Psychology, Ryerson University, Toronto, ON, Canada

**Keywords:** remote research methods, online testing, COVID-19, videoconferencing, infancy, parent–child interaction, zoom

## Abstract

The closure of in-person laboratories and decreased safety of face-to-face interactions resulting from the COVID-19 pandemic jeopardized the ability of many developmental researchers to continue data collection during this time. Disruptions in data collection are particularly damaging to longitudinal studies, in which the testing of different age groups occurs on a continuous basis, and data loss at one time point can have cascading effects across subsequent time points and threaten the viability of the study. In an effort to continue collecting data for a longitudinal study on emotion development started in-person pre-pandemic, we adapted two parent-infant interaction tasks (free-play task and toy removal task) for a remote testing framework. Our procedure for pivoting these tasks to a supervised, remote online testing framework is outlined and the associated strengths and challenges of testing in this format (e.g., feasibility and implementation, testing environment and task setup validity, and accessibility, recruitment, and diversity) are critically evaluated. Considerations for applying this framework to other behavioral tasks are discussed and recommendations are provided.

## Introduction

With the onset of the COVID-19 pandemic and university closures around the globe, developmental researchers largely found themselves forced to move their work activities to a remote platform. Among the many different tasks of a developmental researcher, one activity posed especially difficult challenges in the pivot to the new remote setup: data collection. Although some researchers had previously developed protocols for online testing with developmental samples (e.g., the Lookit platform; Scott and Schulz, 2017; TheChildLab.com; Sheskin and Keil, 2018), the pandemic sparked a widespread need to embrace remote testing as one of the only viable options to continue collecting data (for an example of a collaborative initiative for online testing founded during the pandemic, see ChildrenHelpingScience.com).

One particular context in which disruptions in data collection can have cascading and enduring effects is the collection of longitudinal data. In a longitudinal study, research questions are designed on the premise of having follow-up data at each time point; successful data collection is contingent upon being able to continuously test participants as they “age in” to each brief window of eligibility for participation. When working with infants, these windows of eligibility may be as short as 1–2weeks, depending on the age requirements for participation. Losing data during the follow-up time points of a longitudinal study can render previous years’ worth of data collection and countless time offered up by families unusable. In these cases, there is an obligation to numerous participating bodies to find a way to continue a given research project. There is an obligation to the families who offered their time in the hopes of contributing to the scientific research outlined to them when they consented to enroll in a study; there is an obligation to funding agencies, who provided funds and entrusted the researcher to carry out the proposed work to completion; and on a more personal level, researchers may feel an obligation to the many different laboratory personnel who dedicated their time to helping a study run smoothly over the years, some of whom may be relying on completion of longitudinal data collection for training milestones (e.g., dissertation). The continuity of longitudinal data collection was the primary motivation behind our laboratory’s development of a novel protocol for online testing.

Our longitudinal study was a multi-method study on emotion development across the first 2years of life. Infants were tested at 3.5, 7, 12, and 18months. At 3.5 and 7months, tasks included the still face paradigm (Tronick et al., 1978), ERP, a free-play interaction, and eye tracking, and at 12 and 18months, tasks included eye tracking and a parent-infant toy removal task (Stifter and Braungart, 1995; for more detailed descriptions of the larger study see Segal and Moulson, 2020a, Segal and Moulson, 2020b, and Segal et al., 2021). Data collection began in May 2017, and when testing was shut down in March 2020, we had collected data for 78% of our target sample at 3.5months, 50% at 7months, 29% at 12months, and 21% at 18months. Many aspects of our longitudinal study were not amendable to the switch to online testing; however, we decided to resume testing in October 2020 by adapting two of our parent-infant interaction tasks for remote testing.

This report will outline how we adapted these two tasks, a toy removal task and a free-play task, for online testing. In contrast to preexisting frameworks for remote, unmoderated testing (e.g., Scott and Schulz, 2017; Rhodes et al., 2020), the current framework outlines a method of supervised remote testing in which the researcher is available to guide families through the testing procedures in real-time. This report does not present an empirical comparison across methods; rather, we present areas of considerations for researchers who may be in the preparation or planning stages of moving an in-person task to online testing. There are few guidelines available detailing this process, so our goal is to highlight methodological considerations that may be applicable to the adaptation of other behavioral tasks, beyond the two tasks presented in this paper.

## Materials and Methods

### Task Descriptions

#### Toy Removal Task

The toy removal task (Stifter and Braungart, 1995) was designed for measuring emotion regulation in infants, as it simulates a routine frustration-eliciting situation and provides an opportunity for researchers to measure regulatory behaviors. Our instantiation of this task consists of four phases: (1) play (1.5min): parents and infants are provided with a toy and they are instructed to play together; (2) toy removal (2min): parents are instructed to take the toy away and place it somewhere out of reach but still within sight of the infant. Parents are requested to refrain from speaking to or touching the infant during this time and may be provided with materials to help keep their attention directed away from their infant (e.g., questionnaire or magazine); (3) parent attention return (1min): without returning the toy, parents are permitted to resume interacting with the infant as normal (e.g., talking and touching); and (4) toy return (1min): parents are prompted to return the toy to the infant and to resume playing together.

#### Free-Play Task

This task consists of a 10-min free-play interaction between parents and infants. Parents are instructed to play with their infant as they normally do at home, and they are permitted to use any toys available to them. The interaction is video recorded.

### Online Testing Procedure

Both tasks were run synchronously during an online testing session guided by a researcher over Zoom (Zoom Video Communications Inc., San Jose, CA, United States). Families who had previously participated in our longitudinal study and whose infants were approaching eligibility for the 12- or 18-month time points were contacted and provided information about the online continuation of the study. Parents were sent a consent form to review, and interested parents were provided with the option to either send back a signed consent form prior to the visit or to provide verbal consent during the testing session. To ensure consistent use of the same toy across participants during the toy removal task, a busy box toy (VTECH Busy Learners Activity Cube) was sent to families in advance using Amazon Prime shipping service. Parents were instructed not to open the toy until the testing session to ensure that it remained equally novel to each infant at the start of the task. Parents who indicated that they had a printer at home were asked to print the Depression Anxiety Stress Scale-21 (DASS-21; Lovibond and Lovibond, 1995) ahead of time (to serve as a distraction for parents during the toy removal phase), and parents who were unable to print it were reassured that the researcher would complete it with them during the testing session. Parents were instructed not to complete the questionnaire ahead of time, as this questionnaire is sensitive in nature, and we preferred that it be completed in the presence of a researcher with clinical training to allow for debriefing.

During the testing session, the researcher first collaborated with the family in finding an optimal setup for the task within their homes, which consisted of the infant and parent seated beside each other at a table with the infant seated in a high chair. An important consideration included assisting the family in finding a location with sufficient lighting to see the infant’s face during the recording (e.g., avoiding backlighting). The researcher reviewed the consent form and task instructions with families. When reviewing the instructions, parents were told that a tone would be played to indicate when to move into each phase of the toy removal task, and the researcher previewed the tone for parents. Parents who were unable to print the DASS-21 prior to the visit were instructed to keep a magazine, book, or their phone nearby to use during the toy removal phase in place of the questionnaire. For parents who opted to provide verbal consent, a standardized consent agreement was pasted into the Zoom chat, and the parent was asked to read it aloud after the recording began. The researcher started recording the task within Zoom and turned off their video and microphone for the duration of the task. During the task, the researcher observed the interaction and timed each task phase, ensuring that the tone was audible for each phase transition (i.e., briefly unmuting to play the tone). If parents required assistance during the task, the researcher was available to guide the parents through task-related or technology-related issues. The task was ended early in cases where infants exhibited consistent crying for greater than 20s, which was the same criterion applied during in-person testing. After the task was completed, the researcher stopped the Zoom recording.

The free-play task was completed directly following completion of the toy removal task. The dyad was given a short break while the researcher explained the rationale, instructions, and required setup for the free-play task. Parents were instructed to bring the camera to a location in their home containing the toys with which the infant typically plays and to set it up so that the entire scene was viewable (i.e., full body of both participants). The researcher asked the parent to orient the infant to be facing the camera when possible (to allow for later facial coding) and provided them with the instruction to play as they normally do at home for 10min. The researcher started a second recording and turned off their camera and microphone once again. After 10min, the researcher stopped the recording. In the case of participants who did not have access to a printer and were therefore unable to complete the DASS-21 questionnaire during the first task, the researcher shared the questionnaire on their screen and completed it with the parent virtually. After completion of the free-play task (and DASS-21 questionnaire when necessary), families were debriefed and given a chance to ask questions about the study.

The toy that participants received in the mail for the toy removal task (VR VTECH Busy Learners Activity Cube) also served as participants’ compensation for participating in the study, as families were given the toy to keep after participation, and it was of similar value to previous in-person monetary compensation.

## Discussion

In this report, we outlined the methodology of two behavioral interaction-based tasks adapted for online testing with infants between 12 to 24months. Guidelines detailing how to adapt in-person tasks for online testing are scarce, and as researchers increasingly embrace remote testing, the development of frameworks designed to help researchers make this transition is well-timed. Our goal is to provide an overview of the adaptations required to modify these tasks for online testing to critically evaluate the strengths and challenges of collecting data in this format. Our considerations may not be applicable to all parent–child interaction tasks, but our hope is that our general approach of adapting the two tasks described above may serve as a starting framework for other researchers interested in adapting other behavioral tasks for online supervised testing (e.g., still face paradigm, book reading tasks, and social touch tasks).

### Feasibility and Implementation

#### Strengths

This supervised, synchronous format for online testing proved to be highly feasible, easy to implement, and presented a number of advantages for data collection with infants. Online testing sessions often require fewer research personnel and are shorter in duration compared to laboratory-based testing. For example, online testing eliminates the time required for setting up the physical laboratory space prior to the family’s arrival, and critically, the time that is required for infants to get acquainted with the testing environment. In our online study, only one experimenter was required to be online with the family for testing (compared to the addition of a research assistant during in-person testing), and the online session was 30min in duration compared to 1.5h when run in the laboratory. Although this discrepancy in duration was partly due to restrictions in what we were able to include in the online assessment (e.g., no inclusion of heart rate measurement during the toy removal task in the online visit), it also reflected a reduction in the time required for infants to become comfortable prior to testing, which tends to be a big source of individual variability in testing times. Online, with families participating from the comfort of their own homes, this “warm up” time is not required, and in the case of our study, the first task typically began within the first 5min of the session.

The ease of recording the tasks directly through Zoom is another factor that contributed to the high degree of feasibility and easy implementation of this online testing format. In contrast to technological difficulties that may arise when using video cameras (e.g., uncharged at the time of testing and missing memory cards), recording the tasks through Zoom was highly dependable. When running behavioral tasks, having high quality video recordings are imperative for later analysis. Video recordings are a critical tool in developmental research, as they enable later coding of rich behaviors that may be fleeting in person, they capture the context in which a behavior is embedded (Adolph, 2020), and from an open-science perspective, they allow for widespread data sharing and reproducibility (Gilmore and Adolph, 2017). With the experimenter available to provide live guidance regarding ideal camera angles and lighting, Zoom appears to be a sufficient method for collecting high quality recordings of parent–child interactions. Furthermore, it is a user-friendly technology with which many people are already familiar. Thus, for both research personnel learning to run the online session, and parents participating in the study, there is a minimal learning load from a technology perspective.

We achieved a high rate of task completion for the online study (31/33 to date; 94%, compared to 61/71 for in-person testing; 86%), which may be related to infants’ increased comfort in their homes compared to the unfamiliar laboratory environment, reduced overall testing time, and the reliability of Zoom for capturing video recordings.

#### Challenges and Recommendations

Although the online testing sessions were conducted with a high degree of ease, there are also a number of challenges associated with this format, as well as considerations that will vary depending on the task being adapted. First, not all components of a study will be amenable for remote testing, including the use of specialized technologies like EEG and ECG, which will limit the types of studies researchers can run and the continuity between data collected in person and remotely. Regarding materials, if running a behavioral task is contingent on a specific item (e.g., consistency of the toy across participants is crucial for the validity of the toy removal task), researchers must find a way to mail or drop off materials to families, which may be more or less difficult depending on the location of the research group and other circumstances. Unanticipated issues may arise with the mailing process outside of the researcher’s control (e.g., shipping delays, supplier running out of stock, and price increases in the middle of a study). Researchers should have a backup plan for getting any required materials to participants prior to starting data collection. Additionally, in our study, the required material was a fun and exciting toy, which made it appropriate to serve as participant compensation as well. In the case of other tasks where the provided materials would not be well suited to serve as compensation, researchers should consider the added cost of sending materials to families in addition to the funds previously set aside for participant compensation.

Additionally, whereas it is easier to set up multiple camera angles for in-person testing, the reliance on Zoom for all recordings limits the different viewpoints available for recording. For the toy removal task, the single recording is sufficient and closely resembles the video recordings from in-person testing; however, the free-play task would benefit from an additional “birds-eye” vantage point, which we are able to capture in the laboratory. Different tasks and coding requirements may be more or less amenable to a single viewpoint recording, which should be considered when deciding whether a behavioral task may be appropriate for online adaptation.

Furthermore, information security and participant privacy are important consideration in adapting tasks for online data collection and data storage. Researchers must take precautions to minimize data breaches, which should be coordinated with their respective research ethics board to ensure compliance with institutional guidelines. For example, the use of Zoom as a platform for conducting and recording sessions was approved by our research ethics board as a secure option for collecting data, and recordings were immediately transferred to a secure server for storage. Researchers should also consider whether they can conduct the sessions from a private location when booking sessions (e.g., where others will not be able to see or hear the session) and have the ability to enable a waiting room feature in the video session to ensure unknown persons cannot join the call. These considerations will help ensure participant privacy, confidentiality, and information security.

### Testing Environment and Task Setup Validity

#### Strengths

Su and Ceci (2021) have highlighted that remote online testing from home includes a trade-off between ecological validity and environmental control, which parallels discussion regarding the tension between “real-world or the lab” testing in psychology more broadly (Hammond and Stewart, 2001; Holleman et al., 2020). In-person home testing has been a cornerstone of developmental research for decades, as measuring infants’ real-world behaviors has been highlighted as an important endeavor across developmental fields (e.g., locomotion; Adolph, 2019), and it is thought to be optimized during home-based testing compared to exclusively relying on highly structured, laboratory-based tasks. Furthermore, home-based testing allows for the capture of naturalistic interactions in the settings in which they typically occur, which may afford greater opportunity for measuring family dynamics unaffected by being in a new setting or the presence of other research personnel. Although these benefits of in-person home testing may extend to remote testing from home, environmental control is more difficult when families are tested remotely, as there are likely to be differences in participants’ physical living spaces, background noise, and other sources of interference/distraction that are more difficult to minimize when the researcher is not present in the physical space. We argue that in the face of this trade-off, interaction-based tasks that aim to simulate everyday naturalistic interactions between parents and infants are particularly well suited for maintaining their validity during home-based remote testing, *especially* when the format includes live interaction with the researcher. Tight environmental control tends to be less of a concern for interaction-based tasks compared to other forms of developmental research with infants, such as looking time studies or other visual attention-based paradigms, which are more sensitive to the impact of environmental influences. In a synchronous testing framework, the researcher can maintain the integrity of the study design by ensuring a similar *enough* task setup across participants to provide a sufficient amount of consistency across participants, even in the face of individual differences in families’ home environments.

#### Challenges and Recommendations

Although we believe that interaction-based behavioral tasks are particularly resilient to the lack of tight environmental control obtainable *via* remote online testing, the decision to move to a remote framework may be task dependent. Researchers will need to consider the degree to which completing the task in a naturalistic, yet uncontrolled environment may be an added benefit or detriment to the task validity. For example, in an emotion regulation context, infant attentional strategies serve as an important regulatory strategy (e.g., scanning the room, shifting attention to a novel object, and maintaining gaze on the desired object, such as the toy; Stifter and Braungart, 1995). Scanning patterns may differ depending on the infant’s familiarity with their environment (e.g., familiar versus novel room) and the amount of stimulating objects in each environment (e.g., minimalist laboratory testing room compared to a home kitchen full of distractors). Other elements that may introduce a small degree of variability between participants include pets walking into the room during a task, or the noise of other family members in the background. The degree to which these uncontrolled elements impact the validity of a task will depend on the specific behavioral task and serves as an important area of consideration for researchers contemplating moving a task to a remote testing framework. This challenge is similar to what might be encountered with in-person home testing; however, some of these uncontrolled elements may be amplified in a remote framework where the researcher is not on-site to manage some of the environmental differences.

### Accessibility, Recruitment, and Racial and Socioeconomic Diversity

#### Strengths

Online testing greatly improves accessibility. Shorter testing sessions and the elimination of travel made possible through online testing offer greater flexibility with respect to scheduling, which is a critical ingredient in mitigating attrition in longitudinal studies. Our laboratory has previously found it difficult to re-recruit infants in the older age range of our longitudinal study (e.g., 29% attrition between 3 to 7months vs. 51% attrition at 12months and 57% attrition at 18months), which is largely due to parents’ returning to work and reduced availability. These scheduling constraints are further exacerbated by studies with longer testing sessions. Remote online testing offers greater flexibility for evening testing (e.g., less travel time and sessions are less likely to overlap with infants’ bedtimes) and the ability to book back-to-back sessions to accommodate more weekend testing times (e.g., no turnaround time required to clean up and prepare materials between families), which may facilitate parents’ ability to continue their participation in longitudinal studies after returning to work. The elimination of travel, which has been previously identified as a significant barrier to families’ participation in developmental research (Sugden et al., 2015), strongly contributes to the accessibility of online testing. The option to participate remotely may increase accessibility for families who live further away from universities, and for families who have moved over the course of a longitudinal study. These benefits are similar to those offered by in-person home testing; however, remote online testing eliminates the need for travel for *both* the family and the researcher, rendering it even more advantageous for flexible scheduling.

The increased accessibility of online testing may also lead to improvements in recruiting more racially and socioeconomically diverse samples (Rhodes et al., 2020; Sheskin et al., 2020; Su and Ceci, 2021). Psychology research has traditionally oversampled from Western, Educated, Industrialized, Rich, Democratic (WEIRD) populations (Henrich et al., 2010), which threatens the generalizability of research findings and further marginalizes low-income and racial minority populations. The elimination of travel may boost participation among families of lower socioeconomic status for whom travel costs may have been a deterrent to participating in laboratory-based testing, and it provides researchers with the option to recruit outside of their direct geographical location. Families who are new to participating in research studies may also feel more comfortable participating from their own homes for the first time (Sheskin et al., 2020).

#### Challenges and Recommendations

In considering how to maximize a study’s accessibility, researchers should try to minimize the materials families require to be eligible for participation. In our study, the only materials required for participation were a laptop or tablet with Zoom capability and a high chair. Families who did not have access to a printer were given the option of providing verbal consent and completing a questionnaire in real-time with the experimenter, such that printing materials beforehand was not a condition for participation. For our free-play task, families were able to use the toys available to them at home, which was fitting for a naturalistic task like this one. Required materials are important for researchers to consider when adapting tasks to increase the accessibility of participation and to consider ways to minimize the burden on participants to source and provide their own materials.

Regarding recruitment, one way that we maximized participation from previously participating families was by expanding the age range at which they were eligible to participate, which allowed us to capture families that had aged out of our more restricted time range. This adjustment was possible for the current tasks because we did not expect significant differences in performance across our expanded age range; however, for other tasks where significant development might be expected within a short window, expanding the age range to maximize participation may not be possible.

Undoubtedly, online testing introduces a new barrier to participation, the requirement of home internet access, which may be disproportionately lacking among low socioeconomic and racial minority populations and may compound issues of “digital divide” across groups (Haight et al., 2014). For example, lower rates of internet access are reported among households with lower incomes, lower levels of education, and recent immigrants (Haight et al., 2014). Recommendations to promote racial diversity in online studies include tailoring recruitment efforts in line with those found to be effective for the specific group of interest (e.g., non-White groups; Sugden and Moulson, 2015), collecting and reporting detailed demographic data, allocating funds for providing participants with mobile hotspots if needed, and exploring the option of mobile testing laboratories when it is safe to implement face-to-face testing (Lourenco and Tasimi, 2020).

### Conclusion

Remote online testing is likely to prevail as an enduring method for conducting developmental research beyond the pandemic (Su and Ceci, 2021); thus, generating and evaluating options for conducting studies of varied methodologies and appropriate for different age groups in a remote format are of paramount importance for the field of developmental science. In considering the advantages and disadvantages of the remote testing framework outlined here, we propose that this synchronous format of online testing offers a highly feasible and easy-to-implement option for collecting infant behavioral data remotely, in which the reliability and validity of the task setup and quality of the data are largely preserved. This format offers many of the general benefits of remote unmoderated testing, including greater scheduling flexibility and potential for more diverse samples. Further, the added component of live interaction with the researcher provides additional benefits previously unique to face-to-face testing, such as the ability to ensure a consistent study procedure is followed across participants. We suggest that behavioral interaction-based tasks are particularly amenable to this synchronous testing format, and we encourage the adoption of this framework across other behavioral tasks, beyond the two presented here.

## Data Availability Statement

The original contributions presented in the study are included in the article/supplementary material, and further inquiries can be directed to the corresponding author.

## Ethics Statement

The studies involving human participants were reviewed and approved by Ryerson University Research Ethics Board (REB). Written informed consent to participate in this study was provided by the participants’ legal guardian/next of kin.

## Author Contributions

SS: study conceptualization, data collection, and original draft — writing. MM: study conceptualization, funding acquisition, original draft — feedback and edition. All authors contributed to the article and approved the submitted version.

## Funding

This research was funded by the Social Sciences and Humanities Research Council of Canada (#435–2017-1438; awarded to MM), the Ontario Ministry of Research, Innovation, and Science (#ER15-11-162; awarded to MM), and Ryerson University.

## Conflict of Interest

The authors declare that the research was conducted in the absence of any commercial or financial relationships that could be construed as a potential conflict of interest.

## Publisher’s Note

All claims expressed in this article are solely those of the authors and do not necessarily represent those of their affiliated organizations, or those of the publisher, the editors and the reviewers. Any product that may be evaluated in this article, or claim that may be made by its manufacturer, is not guaranteed or endorsed by the publisher.
